# The distinctive mechanical and structural signatures of residual force enhancement in myofibers

**DOI:** 10.1073/pnas.2413883121

**Published:** 2024-12-16

**Authors:** Anthony L. Hessel, Michel N. Kuehn, Bradley M. Palmer, Devin Nissen, Dhruv Mishra, Venus Joumaa, Johanna K. Freundt, Weikang Ma, Kiisa C. Nishikawa, Thomas C. Irving, Wolfgang A. Linke

**Affiliations:** ^a^Institute of Physiology II, University Hospital Muenster, University of Muenster, Muenster 48149, Germany; ^b^Department of Molecular Physiology and Biophysics, University of Vermont, Burlington, VT 05405; ^c^Biophysics Collaborative Access Team, Department of Biology, Illinois Institute of Technology, Chicago, IL 60616; ^d^Department of Biological Sciences, University of Northern Arizona, Flagstaff, AZ 86011; ^e^Human Performance Laboratory, Faculty of Kinesiology, University of Calgary, Calgary, AB T2N1N4, Canada; ^f^Heart Center at University Medical Center Göttingen and German Centre for Cardiovascular Research, Partner Site Lower Saxony, Göttingen 37075, Germany

**Keywords:** elasticity, X-ray diffraction, mouse, ultrastructure, force transmission

## Abstract

In muscle, titin proteins connect myofilaments together and are thought to be critical for contraction, especially during residual force enhancement (RFE) when steady-state force is elevated after an active stretch. We investigated titin’s function during contraction using small-angle X-ray diffraction to track structural changes before and after 50% titin cleavage and in the RFE-deficient, *mdm* titin mutant. We report that the RFE state is structurally distinct from pure isometric contractions, with increased thick filament strain and decreased lattice spacing, most likely caused by elevated titin-based forces. Furthermore, no RFE structural state was detected in *mdm* muscle. We posit that decreased lattice spacing, increased thick filament stiffness, and increased non-cross-bridge forces are the major contributors to RFE. We conclude that titin directly contributes to RFE.

## Significance

Residual force enhancement is a fundamental skeletal muscle property where more force is produced after an active stretch than if purely activated at the longer length and is key to jump, locomotive, and stabilizing movements. However, the molecular mechanism is elusive. We demonstrate that the titin protein is the main regulator of residual force enhancement (RFE) by structurally changing the sarcomeric motor proteins in such a way that force production and force transmission are improved after an active stretch, compared to a pure isometric contraction at the long length. Our data provide important functional evidence of titin’s critical role to RFE in situ and support a paradigm shift to the textbook knowledge of force augmentation during physiologically relevant muscle action.

Animal movement through complex environments requires unique mechanical features of not only isometric or shortening (concentric) muscle contractions but also lengthening (eccentric) contractions (1). Eccentric contraction produces immediate and rapid force enhancement at the level of the sarcomere that is greater than that possible during isometric or concentric contractions at the same sarcomere length and level of activation (2). However, force change caused by muscle length change is poorly described by current muscle models (3). Force enhancement improves functional tasks such as countermovement jumps (4), downhill braking (5), and joint stabilization when negotiating complex terrain (6). Clinically, eccentric-focused training accelerates muscle hypertrophy above conventional training and is widely used for athletes and for restoring mobility independence to older adults (7, 8).

**Fig. 1.**
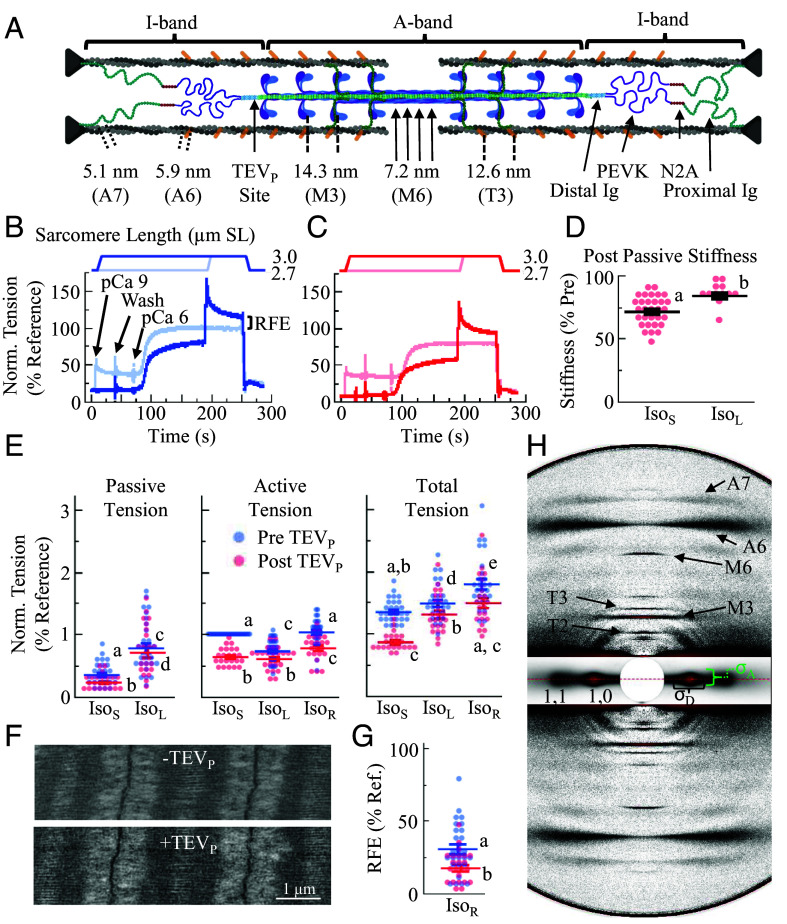
Mechanical properties of TC fiber bundles before and after 50% TC during Iso_S_, Iso_L_, and Iso_R_ conditions. (*A*) Schematic of skeletal half-sarcomere with relevant structural periodicities, I-band titin segments, and TEV_P_ cleavage site indicated. (*B* and *C*) Tension traces of fibers during mechanical experiments before (*B*) and after (*C*) 50% TC, normalized to the total tension during ISO_L_. Active tension is total tension during contractions, minus the passive tension at those lengths. (*D*) Passive stiffness after TC (*n* = 12-31), normalized to its paired pre-stiffness value at Iso_S._ (*E*) Tension before (blue) and after (red) TC for passive, active, and total conditions (*n* = 23-28). Tension is normalized by individual to the active tension during Iso_Short_. (*F*) Transmission electron micrographs of TC sarcomeres after full mechanical protocols with sham (−TEV_P_) or treatment (+TEV_P_) conditions. (Scale bar, 1 µm.) Expanded image available in *SI Appendix*, Fig. S2. (*G*) RFE in fibers before and after titin treatment (*n* = 23-27). RFE is calculated as active tension during ISO_R_—active tension during ISO_L_ and normalized to active tension during ISO_S_. (*H*) Representative X-ray diffraction pattern of skeletal psoas fibers during Iso_S_ before TC, with labeled reflections indicating relevant periodic structures that are referenced in (*A*). An X-ray pattern from a sample after TEV protease treatment is presented in *SI Appendix*, Fig. S3. Statistics: ANOVA design with random effect of individual, followed by Tukey HSD multiple comparison procedure on significant main effects (*P* < 0.05). Data displayed as connecting letters: Different letters are significantly different (Tukey HSD *P* < 0.05). Data throughout reported as mean ± SEM. Full statistical details in *SI Appendix*, Table S1.

During eccentric contraction, both cross-bridge and non-cross-bridge structures are stretched and produce viscoelastic forces that contribute to force enhancement (9). It has been suggested that cross-bridge-based force enhancement plateaus after a short stretch of ~28 nm per sarcomere (9, 10), while further stretch increases force via non-cross-bridge, viscoelastic “spring” elements that increase stiffness upon activation (9, 11, 12). After stretch, force enhancement from cross-bridges dissipates quickly while the non-cross-bridge elastic component remains, leaving a long-lasting enhancement of force above that of pure isometric contractions at the same final length, the so-called residual force enhancement (RFE) (13). The mechanisms underlying RFE are unclear but mounting evidence suggests that the titin protein plays a key role. Titin, the largest known protein, extends from the Z-disk to the M-band, where it runs along the thick filament in the A-band, and is extensible in the I-band (Fig. 1*A*). Titin extends during sarcomere stretch and becomes 4 to 6 times stiffer upon Ca^2+^ activation (14–18) via yet unresolved mechanisms (19). Increasing titin-based forces during passive stretch (20) and submaximal activation (21, 22) also increases the Ca^2+^ sensitivity of force with increasing sarcomere length. Changes in contraction force or stiffness during RFE are often assumed to be due to changes in cross-bridge recruitment, although this notion has little experimental support (23, 24) with even evidence for cross-bridge detachment during the eccentric stretch (25). Measured fiber force and stiffness after skeletal muscle stretch are the net result of multiple processes occurring simultaneously, including cross-bridge cycling, other in-parallel force-bearing elements (e.g., titin), and other elements in the complex force transmission pathway, making it difficult to separate cross-bridge and non-cross-bridge contributions to force and stiffness.

We investigated titin’s function during skeletal muscle contraction using small-angle X-ray fiber diffraction to track structural changes before and after cleavage of 50% of elastic I-band titin in mouse fast-twitch muscle fibers, and in fibers from “muscular dystrophy with myositis” (*mdm*) mice which lack force enhancement (26). Specific cleavage of I-band titin was achieved by using heterozygotes of a transgenic “titin cleavage” (TC) mouse model (*SI Appendix*, Fig. S1), in which titin is controllably cleaved via an embedded tobacco etch virus protease (TEV_P_) cleavage site (17, 27), which allows for a repeated-measures statistical analysis that accounts for intersample variation and increases power. *Mdm* carries a complex titin mutation that manifests as a small deletion in the titin gene (28, 29). Our results demonstrate that during RFE, in addition to increased tension, there are structural-based X-ray diffraction signatures that are distinct from those of fixed-end isometric contraction. TC attenuates RFE tension, blunts RFE-associated diffraction signatures, and increases disorder in the myofilament lattice. We further report that when RFE is absent, as in *mdm* mice, the structural signature of RFE is also absent. Collectively, we posit that RFE is caused, in part, by decreased lattice spacing, stretched intermyofilament bridge elements (e.g., titin, myosin-binding protein C [MyBPC]), and increased thick filament stiffness, with titin a major regulator of each.

## Results and Discussion

### TC Reduces Mechanical Stability.

We conducted RFE experiments on permeabilized heterozygote TC psoas fibers before and after 50% TC via TEV_P_ incubation. Previous studies presented visual evidence that cleavable titins are distributed ~evenly throughout the fiber (17). We assume that on average 3 of the 6 titins per half thick filament are cleaved, with each half-thick filament having between 0 and 6 cleavable titins, randomly distributed throughout the fiber. Due to titin’s important role in sarcomeric integrity, we previously reported that contracting TC fibers with 50% titins cleaved at pCa 4 (maximum activation) quickly degrades the sarcomere (17). However, contractions of titin-cleaved fibers at pCa 6 (~50% maximum tension) did not lead to this degradation, as observed with transmission electron microscopy of fibers after the experiments below (*SI Appendix*, Fig. S2), allowing for these critical contraction experiments. X-ray diffraction patterns and force traces were obtained under three conditions before and after 50% TC (Fig. 1 *B* and *C*): isometric contraction at 2.7 μm sarcomere length (SL) (short isometric contraction; IsoS), isometric contraction at 3.0 μm SL (long isometric contraction; IsoL), and stretch-hold contraction from 2.7 to 3.0 μm SL (isometric contraction after an active stretch [RFE]; IsoR). RFE under submaximal activation conditions (pCa < 5) displays properties such as length-dependent activation (e.g., length dependence of calcium sensitivity) (30). One study (31) generated calcium sensitivity curves for Iso_L_ and Iso_R_ states and found that the pCa at half-maximum active tension (pCa_50_) was larger in the Iso_R_ condition, indicating an increase of calcium sensitivity. Another study (32) studied RFE in rabbit psoas fibers at pCa 4.5 and pCa 6.0, where it was observed that the %RFE over the reference length was 2.6-fold larger at pCa 6.0 than at pCa 4.5. Both studies’ results could be accounted for if myosin head participation increased. These hypotheses were assessed more directly in the current paper using the X-ray diffraction method, where a change in myosin head participation would be identifiable (see below).To evaluate RFE rundown from repeated experiments not related to TC, we conducted sham experiments without the treatment and found no statistical change from the first to second set of experiments (*SI Appendix*, Fig. S3).

We next conducted these experiments before and after 50% I-band TC. Compared to pre-titin cleavage, post-cleavage fibers produced less tension, but the shape of the tension traces was similar (Fig. 1 *B* and *C*). For Iso_S_ and Iso_L_, 50% TC reduced passive stiffness (measured here by sinusoidal oscillations; Fig. 1*D*), passive tension, active tension, and total tension (*P* < 0.01, Fig. 1*E* and *SI Appendix*, Table S1), but the relative difference after treatment was always greater for Iso_S_ vs. Iso_L_ (*SI Appendix*, Fig. S4). Titin-based force and stiffness are known to be critical for thick filament centering in the A-band, with greater stability at longer SLs where titin-based forces are higher (17, 33). Indeed, at a near-slack SL of 2.4 μm, the A-bands of titin-cleaved fibers fell apart during maximal activation (pCa 4) (17), but were maintained here at longer SLs and at submaximal activation (pCa 6; Fig. 1*F* and *SI Appendix*, Fig. S2). Furthermore, RFE (measured as the increase in total force from Iso_L_ to Iso_R_) was reduced after TC from 30.7 ± 3.4% to 17.4 ± 2.2% of reference force (Iso_L_) (paired *t* test; *P* < 0.01; Fig. 1*G* and *SI Appendix*, Fig. S4), indicating that intact titins play a meaningful role in the generation of enhanced force above the reference value. RFE was then converted to force per thick filament (f_TF_), assuming that all forces are transmitted to the thick filament and that in samples with a d_1,0_ = 37.69 nm, ~80% of the cross-sectional area is occupied by myofibrils, resulting in a thick filament density of ~488 × 10^6^ thick filaments/mm^2^. The active tension increased from 48.26 kPa in Iso_L_ to 68.53 kPa in Iso_R_, resulting in f_TF_ values of 98.95 pN and 140.51 pN for Iso_L_ and Iso_R_, respectively. Therefore, compared to Iso_L_, Iso_R_ increased by 41.56 pN. After titin cleavage, f_TF_ were 60.36 and 77.18 pN for Iso_L_ and Iso_R_, respectively. Therefore, we estimate an increase of 16.82 pN, or ~40.5% the pre-cleavage values. These results demonstrate a direct relationship between I-band titin and RFE.

### Lattice Structure and Order During Iso_R_ Are Structurally Distinct.

**Fig. 2.**
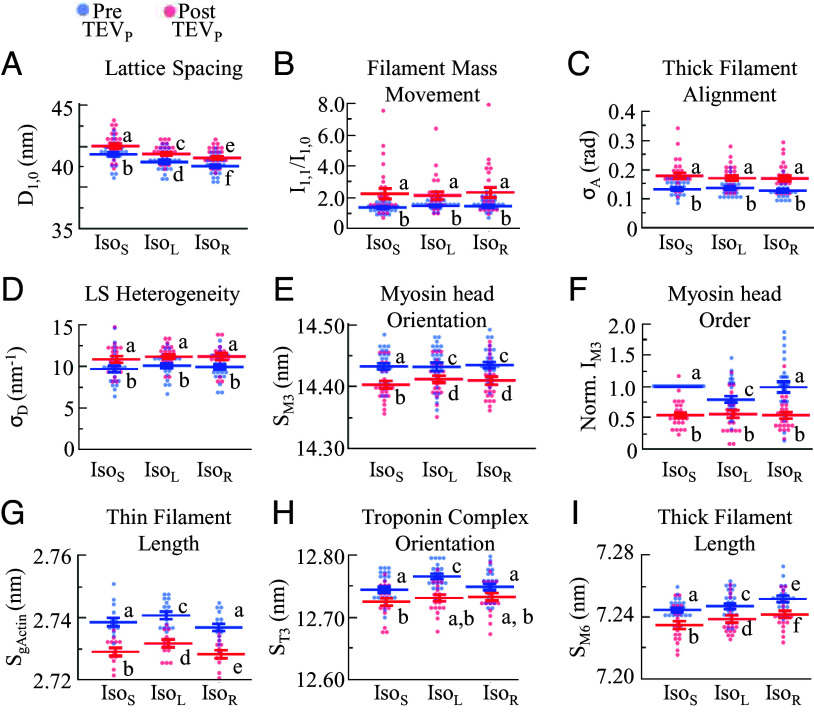
Sarcomeric structural parameters of TC fibers before and after 50% TC. D_1,0_ (*n* = 25-27) (*A*), I_1,1_/I_1,0_ (*n* = 25-27) (*B*), σ_A_ (*n* = 24-26) (*C*), σ_D_ (*n* = 23-25) (*D*) S_M3_ (*n* = 24-26) (*E*), I_M3_ (*n* = 24-26) (*F*), S_gActin_ (*n* =12-16) (*G*), S_T3_ (*n* =19-24) (*H*), and S_M6_ (*n* = 20-25) (*I*) were recorded before (blue) and after (red) 50% TC, at three conditions: Iso_S_, Iso_L_, and Iso_R_. Statistics: ANOVA design with random effect of individual, followed by Tukey HSD multiple comparison procedure on significant main effects (*P* < 0.05). Data displayed as connecting letters: Different letters are significantly different (Tukey HSD *P* < 0.05). Full statistical details in *SI Appendix*, Tables S2 and S3. Intrasample pre–post differences in *SI Appendix*, Fig. S5.

We assessed structural changes using small-angle X-ray diffraction (Fig. 1*H* and *SI Appendix*, Fig. S5), which provides information concerning the underlying structural arrangements of sarcomeric proteins (Fig. 1*A*). We compared diffraction patterns from pure isometric contractions at two different sarcomere lengths (Iso_S_ vs. Iso_L_) and contractions with ramp stretch-hold from the short to the longer length (Iso_L_ vs. Iso_R_). Lattice spacing (LS) was calculated as the separation of the 1,0 equatorial reflections from thick filaments (D_1,0_ plane; Fig. 1*H*). Computational modeling studies suggest that increasing LS decreases force production and blunts length-dependent activation (LDA) by affecting cross-bridge kinetics (34). LS is controlled in part by radial forces imposed on the myofilament lattice by titin (35, 36) and expands in relaxed fibers after TC (22). Furthermore, myopathic human fibers with abnormally stiff titins have smaller LS than controls (37). We report that LS decreased from Iso_S_ to Iso_L_, as expected, but decreased further in Iso_R_ (*P* < 0.0001; Fig. 2*A* and *SI Appendix*, Fig. S6 and Table S2). The “excess” lattice shrinkage in Iso_R_, noted previously (38), suggests that radial forces acting on the myofilament lattice are enhanced after active stretch, suggesting increased titin-based forces in Iso_R_ with respect to Iso_L_. LS is an important regulator of cross-bridge kinetics and therefore cross-bridge-based force production (34), especially in permeabilized preparations, where the lattice is generally expanded compared to intact, and shrinks more rapidly upon stretch. Indeed, lattice spacing for intact preparations changes very little in response to stretch [~36 nm (39)] and so seems unlikely to significantly contribute to RFE in vivo. In permeabilized preparations, as done here, compared to Iso_L_, the Iso_R_ lattice was smaller by 1.08% (0.41 nm). Based on previous assessments (34), we estimated a small but significant RFE contribution of ~5.2% force, or f_TF_ ~ 5.14 pN.

### Structural Evidence of Cross-Bridges Suggests No Differences between Iso_R_ and Iso_L_.

We next considered whether more cross-bridges are present during Iso_R_ vs. Iso_L_. Previous reports using structural, mechanical, or ATP consumption assays suggested no differences in cross-bridge recruitment between Iso_L_ and Iso_R_ (9, 23, 38, 40–43). The equatorial intensity ratio (I_1,1_/I_1,0_) is widely used as a measure of the transfer of mass (i.e., myosin heads) from the thick to thin filaments, with increasing I_1,1_/I_1,0_ indicating more myosin heads associated with the thin filaments (44). We found that before TC, I_1,1_/I_1,0_ remained relatively constant across contraction conditions (*P* = 0.18; Fig. 2*B* and *SI Appendix*, Fig. S6 and Table S2). Iso_L_ has less thick and thin filament overlap compared to Iso_S_, implying that I_1,1_/I_1,0_ would be expected to decrease if nothing else changed (42). Therefore, the finding that I_1,1_/I_1,0_ is not significantly different between short and long SL contraction conditions suggests increased cross-bridge recruitment at longer SLs (i.e., Iso_L_ and Iso_R_) to compensate for the effect of decreased thick–thin filament overlap, as expected via myofilament LDA (45). These findings also imply that there is no further cross-bridge recruitment in Iso_R_ vs. Iso_L_, a finding that corroborates other studies using different methodologies (9, 23, 40, 41).

TC by 50% led to an overall increase in I_1,1_/I_1,0_ across contraction conditions, with a large increase in the spread of the values including some with unrealistically large values greater than the rigor state (twofold to threefold; 44) (*P* < 0.0001; Fig. 2*B* and *SI Appendix*, Fig. S6 and Table S2), which was initially puzzling. These results are likely due to increased lattice disorder after TC as evidenced by increased σ_A_, an indicator of spread of the angular orientation of the sarcomeres across adjacent myofibrils (46), and σ_D_, a measure of LS heterogeneity, after TC (*P* < 0.001; Fig. 2 *C* and *D* and *SI Appendix*, Table S2). Because we cannot easily uncouple the effects of lattice disorder (47, 48) and mass shift due to myosin head movement on I_1,1_/I_1,0_, it would be unwise to interpret increased I_1,1_/I_1,0_ after TC as indicating more transfer of myosin heads toward actin. However, in contrast to the equatorial reflections, meridional patterns are less affected by lattice disorder. Myosin head configuration can be evaluated via the spacing (S_M3_) and intensity (I_M3_) of the M3 myosin meridional reflection. Increases in S_M3_ are typically associated with increasing cross-bridge recruitment, or the reorientation of myosin heads in a position that increases the chance of attachment (49). Before TC, S_M3_ varied among conditions as follows: Iso_S_ < Iso_L_ = Iso_R_ (*P* < 0.0001; Fig. 2*E* and *SI Appendix*, Fig. S6 and Table S2), similar to I_1,1_/I_1,0_ before TC, both suggesting increased cross-bridge recruitment at longer SL, but unaffected by previous active stretch. In comparison, TC reduced S_M3_ across all conditions (*P* < 0.0001; Fig. 2*E* and *SI Appendix*, Fig. S6 and Table S2). Decreased S_M3_ across conditions after 50% TC suggests that titin is important in modulating myosin head action not only in passive muscle but also during contraction—a point previously suggested in muscles from mice with titin mutations but never shown by cutting of titin springs within a sample. Additional support for this notion comes from I_M3_, which provides additional details about variation in myosin head orientation. Before TC, I_M3_ decreased from Iso_S_ to Iso_L_ (*P* < 0.001; Fig. 2*F* and *SI Appendix*, Fig. S6 and Table S3), as expected due to decreased filament overlap (49, 50). However, I_M3_ for Iso_R_ > Iso_L_ = Iso_S_ (*P* < 0.001; Fig. 2*F*), even though they are at the same SL, suggesting that the orientation of heads is more ordered in Iso_R_. Of note, after TC, I_M3_ was reduced in all conditions to similar values, suggesting that titin-based forces are partially responsible for myosin head order, and the conditional effect with passive or active stretch. Interpretation of I_M3_ after TC should be done with caution, as the uneven cleavage of titins across thick filaments will lead to axial displacement of thick filaments with respect to its neighbors, which can itself reduce meridional reflection intensities.

The thin filaments also provide structural information indicative of cross-bridge recruitment (51). In passive cardiac and skeletal muscle, thin filaments extend, and troponin complexes reorient, with sarcomere stretch in a way that has been linked to increasing Ca^2+^ sensitivity (20, 22). The A6 (S_A6_) and A7 (S_A7_) spacings report on the left- and right-handed actin helical structures within the thin filament (*SI Appendix*, Fig. S7 and Table S3) and are used here to calculate the axial spacing of the actin monomers (S_gActin_) (52, 53), where S_gActin_ can be used as a measure of thin filament extension (54, 55). Also relevant for this discussion are structural changes in the troponin complex, indicated by changes in the spacing (S_T3_) of the third troponin meridional reflection T3 arising from the troponin complexes spaced every ~38 nm along the thin filaments. We found that before TC, both S_gActin_ and S_T3_ are Iso_S_ < Iso_L_ and Iso_S_ = Iso_R_ (*P* < 0.01; Fig. 2 *G* and *H* and *SI Appendix*, Table S3). Iso_S_ = Iso_R_ is noteworthy because in purely isometric or passive conditions, the thin filament acts as a stiff Hookean spring so that changes in thin filament strain can be directly interpreted as changes in the total (passive + active) force exerted on the filament (22, 51), as observed from Iso_S_ to Iso_L_; Fig. 1*E*). However, while Iso_R_ forces are larger than Iso_S_, the thin filament strains are ~equal. This suggests that something distinct is occurring during eccentric contraction that decouples force and thin filament strain and may suggest that cross-bridge-based force is decreased while non-cross-bridge-based force is increased. An ANCOVA analysis of S_T3_ with S_gActin_ covariate indicates that the S_T3_ change between conditions is coupled to S_gActin_ but is also partially independent of S_gActin_ (*P* < 0.001; Fig. 2*H* and *SI Appendix*, Table S3). The mechanism or physiological relevance of this independent S_T3_ action is unknown.

Increased titin-based forces may not necessarily result in increased thin filament strain because titin’s tether points are far away from the tip of the thin filament; titin’s permanent interaction with the thin filament is close to the Z-disk (56, 57), and other potential alternative interaction sites are at or around the N2A region in the I-band (58, 59). Although a definitive mechanism remains elusive, another possibility is disruption of MyBPC thick-thin bridges during the eccentric phase of the Iso_R_ condition. MyBPC bridges are purported to be important for contraction (22, 60–62) and seem to be forcibly ruptured by a quick passive stretch (22). There are a total of 27 MyBPC molecules across the half-thick filament C-zone (60). Based on a thick and thin filament length of 1.65 and 1.1 µm, respectively (51), ~21 and ~12 MyBPC molecules can participate in the overlap zone at 2.7 and 3.0 µm SL, respectively. Therefore, a role for MyBPC to explain the Iso_R_ seems plausible and could be directly studied using an inducible “cut and paste” MyBPC mouse line (63).

### Thick Filament Strain Suggests That Titin-Based Forces Are Enhanced in Iso_R_ vs. Iso_L_.

Arguably the most contentious debate in the RFE field is whether titin contributes to RFE by producing more force during Iso_R_ vs. Iso_L_. The hypothesis is as follows: Upon activation, titin stiffness increases by 4 to 6 times (15, 17, 18, 23), which would imply that during and after eccentric contractions, titin-based forces would be greater than if just activated at the longer SL. Mechanically, titin-based force pulls on and stretches the thick filament, with increasing titin force extending the thick filament (21, 22, 35, 64). We found that thick filament strain, quantified via the spacing of the M6 meridional reflection (S_M6_), increased progressively as follows: Iso_S_ < Iso_L_ < Iso_R_ (*P* < 0.0001; Fig. 2*I* and *SI Appendix*, Table S2), suggesting higher titin-based force in Iso_R_ vs. Iso_L_. Additionally, 50% TC slightly reduced S_M6_ across all conditions (*P* < 0.0001 for both; Fig. 2*I* and *SI Appendix*, Table S2) but the general relationship among conditions was preserved. These data indicate titin as a key contributor to thick filament strain.

In addition to titin, MyBPC can also apply a pulling force to strain the thick filament. To date, MyBPC plays an unclear role during contraction, but can also function like a spring and store some level of force (62), so storing force during an eccentric stretch seems plausible. On the other hand, MyBPC is relatively short and so would most likely rupture and reattach to the thin filament during the ~150 nm per half thick filament eccentric stretch conducted here, minimizing its contribution to force exerted on the thick filament during Iso_R_, although some contribution cannot be discounted. Apart from MyBPC, cross-bridge forces during contraction also strain the thick filament (64). However, our analyses above, along with other mechanical and biochemical studies (9, 23, 40, 41), suggest no differences in the degree of cross-bridge recruitment between Iso_L_ and Iso_R_, so that the contribution of cross-bridge-based thick filament strain should be similar between Iso_L_ and Iso_R_. Based on our analysis, we postulate that the thick filament strain in Iso_R_ relative to Iso_L_ is primarily due to increased titin-based force after active stretch.

We next used the thick filament strain data to provide estimates of f_TF_ during Iso_R_, which also includes the LS-dependent effects described above. Compared to Iso_L_, the thick filament in Iso_R_ is strained 0.069% more, with 41.6 pN more f_TF_. The excess f_TF_ is a product of both the forces acting to strain the thick filament, such as parallel elastic elements (e.g., titin and possibly MyBPC), and the changes in force that arise primarily from stiffening of the thick filaments when they are stretched (65, 66). Approximating changes to thick filament stiffness (Stiff_TF_) is a nontrivial task, as thick filament stiffness is nonlinear (21). To accomplish this, we derived f_TF_ as a function of percent thick filament elongation (e_p_) using active tension-thin filament strain data from fast twitch mouse muscle (21), and converted to f_TF_ - thick filament strain. The data were described well by a third-order polynomial: f_TF_ = 2.98*10^−8^e_p_ + 709e_p_^3^, simplified to f_TF_ = 709e_p_^3^, with the Stiff_TF_-strain relationship equal to the derivative of f_TF_, Stiff_TF_ = 2127e_p_^2^. We next calculated the e_p_ values for Iso_L_ and Iso_R_ from S_M6_ values as follows: Passive S_M6_ at 3.0 μm SL is 7.205 nm (21, 22, 35, 64), and the Iso_L_ and Iso_R_ S_M6_ = 7.247 and 7.252 nm, respectively. Therefore, from the passive state, the thick filaments are strained 0.586% and 0.655% during Iso_L_ and Iso_R_, respectively. Plugging the e_P_ values into the Stiff_TF_-strain equation produces 729.7 and 912.9 pN/e_p_ for Iso_L_ and Iso_R_, respectively. Therefore, the thick filament stiffness increases by 25.1% from Iso_L_ to Iso_R,_ which also reflects an up to ~25.1% increase in f_TF_ (67). However, the exact relationship between Stiff_TF_ and f_TF_ is somewhat less than linear, depending on the properties of thick filament compliance. Because the relationship is not yet well defined (65, 66), we used a conservative lower bound of ~0.5 Stiff_TF_ to f_TF_ ratio. Said another way, ~12.4 pN up to a maximum of ~24.8 pN of the measured RFE per thick filament (f_TF_ ~ 41.6 pN) can be associated with mechanical stiffening of the thick filament. Of note, during maximal activation (pCa 4), the thick filament is already elongated to the point where further stretch would not further change stiffness appreciably (21) and so we would predict very little extra thick filament stretch in Iso_R,_ and thus a reduction or removal of this contribution to RFE—but this prediction is worth empirical evaluation.

An open question is whether this unique structural state during Iso_R_ is required for any RFE. To assess this point, we utilized the muscular dystrophy with myositis (*mdm*) mouse, which to our knowledge is the only skeletal mouse model in which RFE can be induced in wild-type but not in HOM *mdm* (26, 68). We harvested EDL muscle between 23 and 39 d old to study the muscle with the *mdm* mutation but before signs of sarcomeric disruption become apparent (26, 68, 69). We assessed whether the structural indicators of the RFE state were also missing in HOM *mdm* skeletal muscles (*SI Appendix*, Fig. S8). Experiments were run similarly to the TC experiments, but at different SLs for the three contraction types: isometric 2.4 μm SL (*mdm* Iso_S_), isometric 3.2 μm SL (*mdm* Iso_L_), and RFE (*mdm* Iso_R_; stretch-hold from 2.4 to 3.2 μm SL). Compared to TC fibers before TC, WT *mdm* fibers showed similar diffraction signatures of RFE for LS, S_M6_, I_1,1_/I_1,0_, σ_A_, S_M3_, and S_A6_ (*SI Appendix*, Fig. S8 *A*–*F* and Table S4). In contrast, *mdm* fibers showed no statistical difference in any of these parameters between Iso_R_ and Iso_L_ except for I_1,1_/I_1,0_. Here, as in the TC experiments after TC, HOM *mdm* produced unusually large I_1,1_/I_1,0_, with associated increases in σ_A_, suggesting reduced thick filament stability in HOM *mdm* fibers, and so inflated I_1,1_/I_1,0_ independent of myosin head mass movement (see discussion above). Based on these data, it is reasonable to propose that there is a causative relationship between the distinctive diffraction signature during Iso_R_ and the presence of RFE.

### Titin-Based Forces Help Regulate Myofilament Activation Levels.

Our experiments are conducted at submaximal pCa levels, which align more closely with the in vivo environment where pCa-levels do not reach the maximum (pCa > 5). In these submaximal conditions, the filament activation can be tuned to meet the current demands. Muscle models with modified titin-based forces show that decreasing titin-based stiffness decreases active force, and vice versa (15, 70). However, these models often show a secondary disease state caused by the dysfunction, making it difficult to parse out what the titin modification is doing, compared to the ensuing disease state. Here, we were able to modify perceived titin-based stiffness and forces in healthy muscles by TC, and indeed, our mechanical (Fig. 1) and structural data (Fig. 2) provide clearest evidence to date that TC leads to a decrease in myofilament activation levels across all conditions tested here. In this study, we were able to modify perceived titin-based stiffness and forces in healthy muscles by TC, and indeed, our mechanical (Fig. 1) and structural data (Fig. 2) provide the clearest evidence to date that TC leads to a decrease in myofilament activation levels across all conditions tested here. These findings are in alignment with (31) who demonstrated that calcium sensitivity (often linked to myofilament activation level) is increased from Iso_L_ to Iso_R_, while cleavage of titin by trypsin reduced calcium sensitivity across both conditions.

While TC reduces the myofilament activation level across conditions, there are a few situations where the magnitudes are different between conditions. Generally, markers for the filament lattice are similarly changed across conditions (e.g., D_1,0_, I_1,1/_I_1,0_, σ_D_, σ_A_), suggesting a simple relationship to titin-based forces. However, myofilament structural features can be more complicated. Most noticeable is a change in the relationship of I_M3_, a marker of head orderness and often used to indicate cross-bridge engagement (increased I_M3_, decreased cross-bridge engagement). In pre-cleaved conditions, heads are more ordered in Iso_L_ vs. Iso_S_ and Iso_R_, suggesting more cross-bridge engagement in Iso_L_ that is somehow lost in the Iso_R_ condition. It is possible the previous stretch in the Iso_R_ is the cause, but further study is needed. Regardless of the reason, in post-cleaved conditions, this conditional effect is lost with samples still presenting ~similar levels of cross-bridge recruitment. Based on magnitude, Iso_L_ proportionally changed the least, and so we speculate that there is a basal level of cross-bridge recruitment caused by calcium that cannot be reduced further, regardless of titin-based regulatory processes. Whether this titin-independent, (presumably) calcium-controlled basal level of cross-bridge recruitment of the myosin heads can itself be tuned, perhaps via phosphorylation of MyBP-C (71) or the essential/regulatory light chain (72), is an interesting avenue of further investigation.

### A Stiffer Titin in Iso_R_ vs. Iso_L_ Contributes to RFE.

**Fig. 3.**
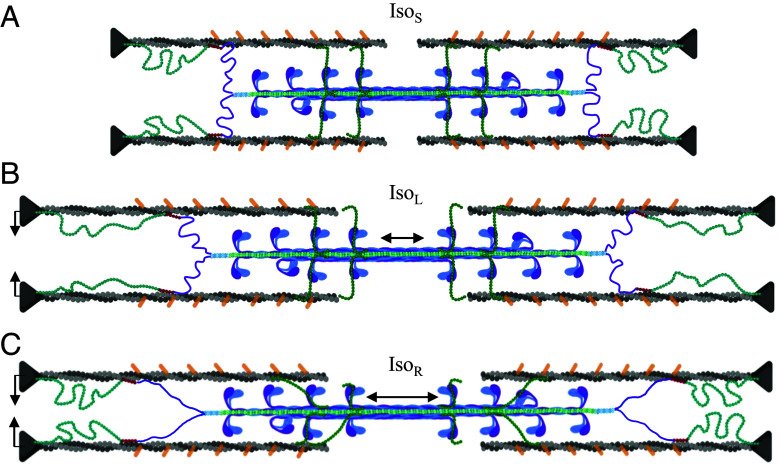
Mechanism of RFE. Configuration of sarcomeric proteins during (*A*) Iso_S_, (*B*) Iso_L_, and (*C*) Iso_R_ that can account for their distinctive mechanical and structural signatures. Filament extension is dramatized for visualization purposes and is smaller in real sarcomeres. In passive muscle, low-level titin-thin filament interactions occur in such a way that passive stretch is enough to detach-reattach and/or drag titin along the thin filament so that titin-based free length and extension are still like if they were not attached at all. During contraction, the titin-thin filament interaction becomes stronger, so that during an eccentric contraction, titin extension occurs above that in passive, producing elevated titin-based force and explaining the mechanical and structural signatures in Iso_R_. Increased titin-based force contributes to RFE, which also leads to smaller lattice spacing and increased thick filament stiffness, improving force production and force transmission, respectively.

The underlying mechanism(s) of RFE, here f_TF_ = 41.6 pN above Iso_L_ tension, have long been unresolved. With this X-ray diffraction dataset, we thus far estimated two mechanisms that contribute to RFE: shrinking LS and increased thick filament stiffness (f_TF_ ~ 12.4-24.8 pN). This implies that there is still ~16.8 to 29.2 pN of f_TF_ unaccounted for. This force most likely comes from the stretch of non-cross-bridge myofilament bridge proteins. Titin is thought to become stiffer upon activation, and we can calculate how much stiffer it would become if it did indeed account for the missing component. On this approximation, we assume that titin contributes all forces imposed on the thick filament and that there are 12 titins per thick filament (6 titins per half-thick filament), equating to ~2.8 to 4.9 pN per titin. Based on previous data (73), passive titin-based forces are ~2.5 and ~5 pN per titin at 2.7 and 3.0 μm SL, respectively, equating to a 2.7 to 3.0 μm SL stretch stiffness (~150 nm stretch per titin) of ~0.016 pN/nm. If we add the excess titin-based forces to the 3.0 μm SL values assuming it is all the unaccounted for f_TF_, then the titin-based stiffness after the stretch would be ~0.035 to 0.049 pN/nm, suggesting that upon activation, titin stiffness increased ~2.2 to 3.1 times compared to the passive state. This value will be somewhat lower if MyBPC or other parallel elastic components are involved. Presently, no molecular mechanism for this activation-dependent increase in titin stiffness is agreed upon, although studies are ongoing (19, 59). One idea (11, 19, 58, thoroughly discussed in ref. 74) relies on evidence of relatively weak titin-thin filament interaction at the N2A and nearby PEVK region in passive muscle and that this binding is stronger in the presence of Ca^2+^. This attachment functionally shortens the titin free-length and allows only the stiffer PEVK region to extend with increasing SL. Fig. 3 presents hypothetical configurations that can explain our data, where titin free-lengths are extended more during active vs. passive stretch, and deserves further empirical study. At present and under an as-yet unclear mechanism, we provide strong evidence that titin-based forces are greater after an active stretch as compared to an isometric contraction at the longer length, leading to structural and mechanical changes that enhance force, which answers the decades-old question as to why RFE exists.

## Limitations

The current technique includes the use of permeabilized fibers, which swells the lattice away from that found in vivo. Often, lattice compression molecules such as dextran are used to return the lattice to physiological levels, but we have observed a dextran-associated TEV_P_ inactivity in this model (22, 75) and other models that use TEV_P_ (76). In principle, solution exchanges between pure solutions for TEVp incubations, and those with dextran for experimentation are possible but there are logistical and technical considerations at the X-ray beamline that make this protocol difficult to implement, but solutions are under development. Increased lattice spacing reduces myosin head order and partially shifts myosin heads toward the ON state, changing absolute values measured here away from intact preparations (39). Nevertheless, there is no evidence to suggest that the directionality of the effects of reduced titin-based forces on the myofilaments is different from in vivo conditions. Furthermore, most studies to evaluate RFE used permeabilized preparations without lattice compression, making our results highly comparable to the field’s broader literature. Identifying a strategy to rapidly cleave titin using intact TC preparations so that pre- and post-cleavage measurements can be taken within a sample, is a long-term goal.

Typical sample compliance during fixed-end contractions, both caused by the mechanics rig and the fiber itself, leads to a slight difference of SLs between Iso_L_ and Iso_R_ of up to ~0.1 µm SL (*Materials and Methods*), which under our experiments could produce an apparent RFE of up to ~14%—a meaningful consideration to the ~30% RFE observed in this study. Current strategies to track and immediately correct SL during RFE experiments, such as with a camera or laser spot-follower device are possible at the single fiber level (77). However, this has proven difficult to implement into the larger fiber bundle preparations within the X-ray diffraction experiments, but solutions are under development.

## Conclusions

Our data demonstrate that titin is a critical regulator of sarcomeric tension, and as such, an essential determinant of RFE. Furthermore, the presence of RFE seems to align with a distinctive structural state that is affected by TC and the *mdm* (titin) mutation. Finally, our analysis provides evidence that the generation of RFE is predominately caused by not only increases to titin-based forces but also via titin’s pull on sarcomeric structures that decrease lattice spacing and increase thick filament stiffness. The next critical experiments should capture the distinctive structural signature of RFE in different muscle types (e.g., slow twitch muscles), at different activation levels, and in whole muscle (intact) preparations. Experiments in intact preparations, however, will require an adaptation of the strategy to rapidly cleave titin.

## Materials and Methods

### Animal Model and Muscle Preparation.

#### TC mice.

Animal procedures were performed according to the guidelines of the local animal care and use committee and approved by the local authorities (LANUV NRW, 81-02.04.2019.A472). HaloTag-TEV [TC (27)] mice were bred and housed at the University Clinic Muenster. Genotyping was conducted by PCR in duplicate using custom primers: 5′cgtggtggcttatcttctagc3′ and 5′ctgttggttcatgcatctcc3′, as previously described (17). Genetically heterozygous adult TC mice (age range, 2 to 6 mo) were humanly killed and psoas muscle immediately extracted for long-term storage and permeabilized (“skinned") at −20 °C using standard glycerol techniques [1:1 rigor: glycerol; rigor contains (mM) KCl (100), MgCl_2_ (2), ethyleneglycol-bis(β-aminoethyl)-N,N,N′,N′-tetraacetic acid (EGTA,5), Tris (10), dideoxythymidine (DTT, 1), and protease inhibitors [Complete, Roche Diagnostics, Mannheim, Germany], pH 7.0]. Samples were shipped to the BioCAT facility on ice for all experimental tests and stored at −20 °C until used. On the day of experiments, psoas muscles were removed from the storage solution and vigorously washed in relaxing solution (composition (in mM): potassium propionate (45.3), N,N-Bis(2-hydroxyethyl)-2-aminoethanesulfonic acid BES (40); EGTA (10), MgCl_2_ (6.3), Na-ATP (6.1), DTT (10), and protease inhibitors (complete), pH 7.0)). Bundles containing 15 to 30 fibers (3 to 6 mm long) were carefully excised and kept in physiological register by tying silk suture knots (sizing 6-0 or 4-0) at the distal and proximal ends of the bundle. Samples were then immediately transferred to the experimental chamber (see below).

#### Muscular dystrophy with myositis (*mdm*) mice.

The Institutional Animal Care and Use Committees at Northern Arizona University and Illinois Institute of Technology approved all husbandry and experimental protocols. Heterozygous mice of the strain B6C3Fe a/a-Ttn *mdm*/J were obtained from the Jackson Laboratory (Bar Harbor, ME) and a breeding colony was maintained to produce wild-type and homozygous recessive mice (*mdm*). Wild-type and *mdm* mice were killed at 24 to 30 d via isoflurane gas overdose confirmed by cervical dislocation. Extensor digitorum longus (EDL) muscles were extracted from killed mice following (78). Early studies suggested that *mdm* muscles undergo cycles of degeneration and regeneration starting at 1 to 2 wk after birth in slow-twitch muscles and at 3 to 4 wk after birth in fast-twitch muscles (69, 79) presented transmission electron micrographs of sarcomeres from 23- to 29-d-old *mdm* EDL (fast twitch) muscles, which showed no evidence of filament disorder or differences in I-band length compared to WT littermates. In this study, we harvested EDL muscle within this same age window when there is no apparent disruption of sarcomere structure. From here, the protocol is the same as in TC mice.

#### Experimental setup rationale.

Predominately fast-twitch muscles are typically used for RFE experiments because they often exhibit large RFE, especially when stretch occurs on the descending limb of the force-length curve, as done here (80). The psoas and EDL physiological operating ranges cover the descending limb, with the psoas operating range extending up to ~3.2 µm SL. Our experimental SL range was chosen to avoid overstretch-related sample damage (81). Active stretch velocity was set at 1.0 µm SL s^−1^ as this velocity is known to produce large RFE reproducibly (82).

### Small-Angle X-ray Diffraction and Fiber Mechanics Apparatus.

X-ray diffraction patterns were collected using the small-angle instrument on the BioCAT beamline 18ID at the Advanced Photon Source, Argonne National Laboratory (83). The X-ray beam (0.103 nm wavelength) was focused to ~0.06 × 0.15 mm at the detector plane, with an incident flux of ~3 × 10^12^ photons per second. The sample to detector distance was set between 2.0 and 3.5 m, and the X-ray fiber diffraction patterns collected with a downstream CCD-based X-ray detector (TC experiments: Mar 165, Rayonix Inc, USA; *mdm* experiments: PCCD 16080; Aviex, New York, USA). For TC experiments, diffraction patterns were captured with 1 s exposure times, while for *mdm* experiments, a series of 25 or 50 X-ray diffraction patterns (10 ms X-ray exposure per pattern over 1 s) were collected and combined. An inline camera built into the system allowed for initial alignment with the X-ray beam and continuous sample visualization during the experiment. Muscle preps were mounted on custom muscle mechanics rigs, as explained previously (38, 22). Sarcomere length (SL) was measured via laser diffraction using a 4-mW Helium-Neon laser. Force baseline was set at slack length. After this initial setup, fiber length changes were accomplished through computer control of the motor. Experiments were conducted at 25 °C. The mechanical rig was supported on a custom-designed motorized platform that allowed placement of muscle into the X-ray flight path and small movements to target X-ray exposure during experiments. Using the inline camera of the X-ray apparatus, the platform was moved to target the beam at different locations along the length of the sample. To limit X-ray exposure of any one part of the preparation, no part of the sample was exposed more than once. Fiber bundle diameter was measured using the inline camera, and physiological cross-sectional area calculated at initial fiber length, with the assumption that the sample was a uniform cylinder longitudinally.

### Experimental Protocols and Analysis.

For TC experiments, sarcomere length (SL) was set to an initial length (~2.7 μm SL; L_0_). Length changes were accomplished by manual or computer-driven means. After attachment, fibers underwent several cycles of rapid sinusoidal oscillations (5 × 30 s, with 30 s rest; 50 Hz, ±5% L_0_) on the relaxed preparation to acclimate the sample to the testing environment and detach any leftover cellular debris (e.g., collagen, vasculature) that would otherwise impact force measurements. Passive forces typically stabilized after 2 to 3 trials. The last oscillation series was used to quantify passive muscle stiffness, defined here as the averaged minimum-to-maximum force change of the last 10 oscillations, divided by the stretch amplitude.

The main mechanical tests consisted of two activation protocols, conducted in random order. From previous studies, we found that contraction at pCa 4 (maximal calcium concentration) quickly degraded the sarcomere structures (17). During pilot studies, we found that reducing the calcium concentration to pCa 6 (~50% maximum active tension) alleviated these issues and kept sarcomeres intact throughout the entirety of our experiments. We further imaged a subset of used preparations via transmission electron microscopy to assess sarcomere integrity, with an example shown in Fig. 1*F* and expanded view in *SI Appendix*, Fig. S2.

For the first protocol, the relaxed fiber bundle was stretched from 2.7 to 3.0 μm SL at 1.0 μm SL s^−1^ and held at that length for 30 s to allow for stretch relaxation. The sample was then moved into a bath of washing solution for 30 s x three washes each, followed by a transfer into activating solution x 2 washes (pCa = 6.0). Active forces rose until reaching a plateaued, maintained for up to 2 min, and then shortened back to 2.7 μm SL and deactivated via 2 solution exchanges with relaxing solution. For protocol 2, the relaxed sample was kept at 2.7 μm SL and transferred into washing for 30 s x 3 exchanges, and then activating solution x 2 exchanges. When active forces reached a plateau, an eccentric stretch was performed from 2.7 à 3.0 μm SL at 1.0 μm SL s^−1^ and held at that length for 60 s to allow for stretch relaxation, followed by an immediate X-ray exposure. The preparation was then shortened back to 2.7 μm SL and transferred into relaxing solution x 2 exchanges. For both protocols, isometric active contraction times were adjusted to be as equal as possible to allow for a fare comparison between the protocols. The order of the two protocols was randomly assigned to each sample. Following the two protocols, the sample was incubated with TEV_P_ for 20 min (100 units acTEV_P_ in 300 μL relaxing solution). After incubation, fibers were rinsed in fresh relaxing solution to remove excess protease, and several sinusoidal oscillations (see above) performed to measure stiffness. X-ray diffraction patterns were collected during isometric contraction conditions at 2.7 and 3.0 μm SL and after the stretch-hold (RFE) condition. To evaluate RFE rundown (decreasing RFE caused generally by repeated RFE experiments), we ran a sham experiment of the above experiment, but with incubation in pure relaxing solution without TEV_P_. We did not find statistical evidence of RFE rundown (*SI Appendix*, Fig. S3).

During pilot experiments, we used laser diffraction to assess fiber shortening during contraction. We paid careful attention to limit changes in sarcomere length during fixed end contractions. We utilized equipment with negligible compliance, performed experiments on the descending limb of the force–length curve where significant passive forces are developed. Further the reduced activation level (pCa 6 = ~50% maximum tension production) limits fiber shortening. In total, we observed that fiber shortening was limited to less than 0.1 µm SL at both SLs during isometric contractions and the differences in SLs between isometric 3.0 µm SL and the stretch-hold at 3.0 µm SL contraction condition up to ~0.1 µm SL. Some apparent force enhancement can occur here up to ~14% but cannot account for the much larger total RFE measured (~30% in WT).

*Mdm* experiments were conducted similarly, but with short and long lengths of 2.4 and 3.2 μm SL, respectively. To visualize the ultrastructure of a subset of samples after experiments, we obtained transmission electron micrographs of relaxed samples at ~2.9 to 3.0 µm SL, as previously described (17).

### X-ray Image Analysis.

X-ray diffraction patterns were analyzed using the MuscleX open-source data reduction package (84). The “Scanning Diffraction” routine was used to measure the angular divergence of the 1,0 equatorial reflection. The routine obtains 2D and 1D radially integrated intensities of the equatorial intensities and then fits Gaussian functions over the diffraction peaks to calculate the SD (width σ, σ_D_) intensity distribution pattern. In this process, the routine obtains the integrated intensity of each equatorial reflection as a function of the integration angle. Gaussian profiles are fit to the projected peak intensities to find the SD of the orientation angle (angle σ; σ_A_) as a measure of the angular divergence of the angle that the sarcomeres in the myofibrils make to the long axis of the preparation. σ_A_ is used as a proxy for interthick filament ordering (46). The “Equator” routine of Muscle X was used to calculate the I_1,1_/I_1,0_ intensity ratio (IR), lattice spacing (LS) between thick filaments, and σ_D_, a measure of the variability in thick filament lattice spacing (a proxy for lattice ordering). Spacings of meridional reflections (S_M3_, I_M3_, S_T3_, S_M6_) and off-meridional reflections (S_A6_, S_A7_) were collected using the MuscleX routines “Diffraction Centroids” and “Projection Traces.” Actin monomer spacing (S_gActin_) was calculated using A6 and A7 spacing data as reported previously (85). Every image provides reflections of different quality, which lead to various levels of Gaussian fit errors for each reflection modeled, which increases the variation in spacings in the dataset. To limit these effects, fit errors >10% were discarded. Positions of X-ray reflections on the diffraction patterns in pixels were converted to sample periodicities in nm using the 100-diffraction ring of silver behenate at d_001_ = 5.8380 nm.

### Statistics.

Statistical analysis was conducted using JMP Pro (V16, SAS Institute Inc., Cary, NC). The significance level was α = 0.05. Response variables included all X-ray parameters. We first built a repeated-measures ANOVA design. Of note, we use repeated-measure designs (for TC experiments, tracking changes after TC within samples; for *mdm* experiments, tracking changes within genotypes [i.e., nested design]) because it is a statistically powerful approach that minimizes the false-positive rate for our experiments. For *mdm* datasets, response variables with <5 data points were excluded from analysis. For TC experiments, we used fixed-effects treatment (pre-/post-TEV_P_ incubation), condition (Iso_S_, Iso_L_, Iso_R_), a treatment x condition interaction term, and a random (repeated-measures) effect of individual. For *mdm* experiments, we used fixed effects genotype (WT/*mdm*) and condition (Iso_S_, Iso_L_, Iso_R_), a genotype x condition interaction term, and a random (repeated-measures) effect of individual nested within genotype. Data were best Box-Cox transformed to meet assumptions of normality and homoscedasticity when necessary, which were assessed by residual analysis, Shapiro–Wilk’s test for normality, and Levene’s test for unequal variance. Significant main effects were subject to Tukey's highly significant difference (HSD) multiple comparison procedures to assess differences between factor levels. These data are indicated in graphs via so-called connecting letters, where factor levels sharing a common letter are not significantly different from each other.

## Supplementary Material

Appendix 01 (PDF)

Dataset S01 (XLSX)

## Data Availability

All study data are included in the article and/or supporting information.
