# Supplementary material for: The distinctive mechanical and structural signatures of residual force enhancement in myofibers
Source: Proc Natl Acad Sci U S A. 2024 Dec 16;121(52):e2413883121. doi: 10.1073/pnas.2413883121 (PMC11670058; doi:10.1073/pnas.2413883121)
Supplement: Supplementary file 1 — Appendix 01 (PDF) [file pnas.2413883121.sapp.pdf]

PNAS

Supporting Information for
The distinctive mechanical and structural signatures of residual force enhancement in myofibers

Anthony L. Hessel*, Michel Kuehn, Bradley M. Palmer, Devin Nissen, Dhruv Mishra, Venus Joumaa, Johanna K. Freundt, Weikang Ma, Kiisa C. Nishikawa, Thomas C. Irving, Wolfgang A. Linke

Corresponding author: Anthony L. Hessel

Email: anthony.hessel@uni-muenster.de

This PDF file includes:

Figures S1 to S8
Tables S1 to S4

Other supporting materials for this manuscript include the following:

Datasets S1

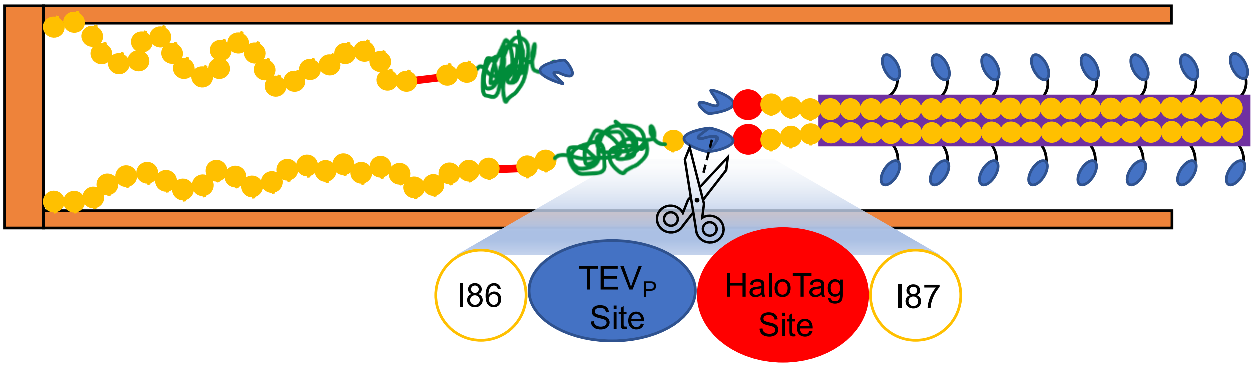

Fig. S1. Titin cleavage (TC) mice allowed for intra-sample assessments of titin function. A clean experimental strategy to study titin function is to specifically cleave I-band titin and terminate its functionality in a targeted and controllable fashion, allowing for changes to be tracked within the same preparation. The titin cleavage (TC) mouse model was generated with a cloned-in HaloTag-TEV cassette inserted into I-band titin close to the A-band (*24*). The Tobacco Etch Virus (TEV) protease recognition site is specifically cleaved by the TEV protease, while the HaloTag domain allows for easy protein labeling, useful for the assessment of titin cleaving. The insertion itself does not affect mouse development, muscle structure, or performance (*17*, *27*) and so allows for the study of as purely a titin-based effect as possible. Figure used with permission from (*22*).

A RFE protocol, no TEV_P treatment

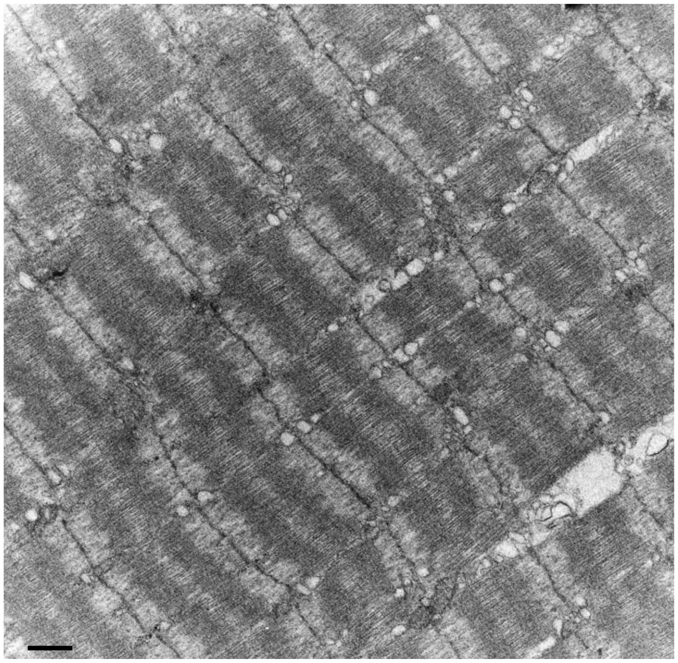

B RFE protocol, TEV_P treatment

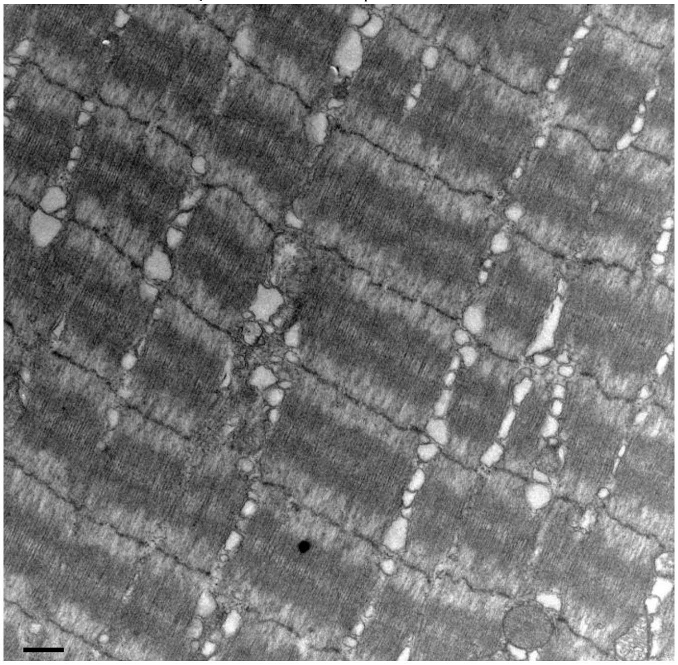

Fig. S2. Representative images of samples after RFE experiments. The complete RFE experimental protocol was run without (*A*) or with (*B*) TEV protease (TEV_P) treatment to assess protocol-associated deterioration of sarcomere structures. After experiments, samples were stretched to ~2.9-3.0 µm SL and prepared for TEM imaging. Both treatment types withstood the protocols without significant sarcomere deterioration. The A-band borders in samples treated with TEV_P are visible – a typical feature if titin is cleaved in passive muscle (*17*, *22*). Scale bars, 1 µm.

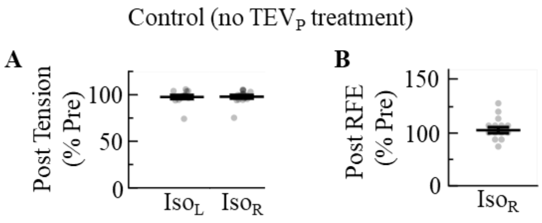

C

Parameter (n)	Mean ± s.e.m. %Initial value	95% Confidence interval
Iso_L (14)	0.97 ± 0.02	[0.93 – 1.02]
Iso_R (14)	0.98 ± 0.02	[0.94 – 1.02]
RFE (14)	1.05 ± 006	[0.93 – 1.17]

Fig. S3. Tension differences after control mechanical experiments. In the TEV_P experiments, each sample was run through all conditions two times, but in this case, no TEV_P incubation was conducted, only a change in relaxing solution. (*A*) The post tension values normalized to the paired pre-values for the passive component and active components during Iso_L, Iso_R (defined in main text). (*B*) The post RFE values normalized to the paired pre RFE values. (C) Descriptive statistics of data in A and B. RFE is the Iso_R of panel B and is the changed in the calculated residual force enhancement of a sample before and incubation in control solution. Data shown as mean ± 95% confidence interval of the mean. The intervals cover 1.0, which indicates that the tension from the second trials is not significantly different from its paired initial trail.

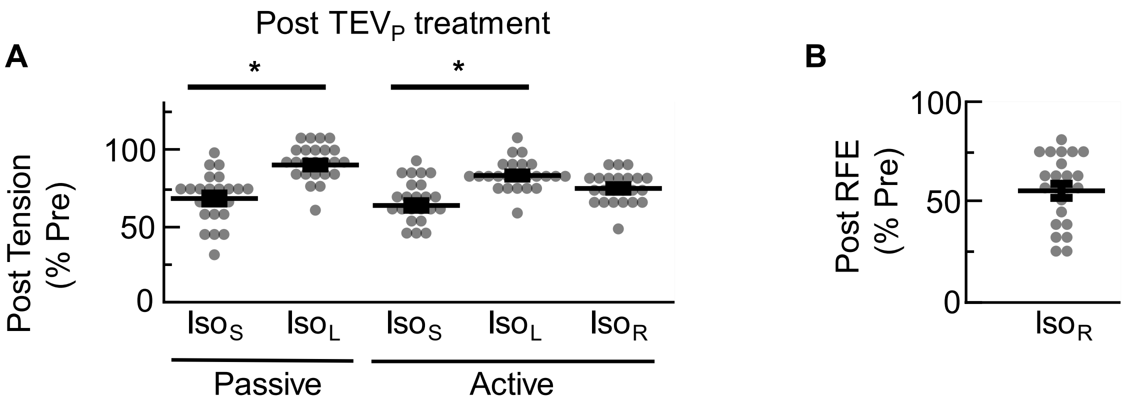

Fig. S4. Tension differences after 50% titin cleavage from mechanical experiments. (*A*) The post tension values normalized to the paired pre-values for the passive component and active components during Iso_S, Iso_L, Iso_R (defined in main text; separate data shown in Fig. 1E). (*B*) The post RFE values normalized to the paired pre RFE values (separate values shown in Fig. 1G) * $P < 0.05$ between the short (Iso_S) and long (Iso_L) conditions, assessed via ANOVA. Data shown as mean ± s.e.m.

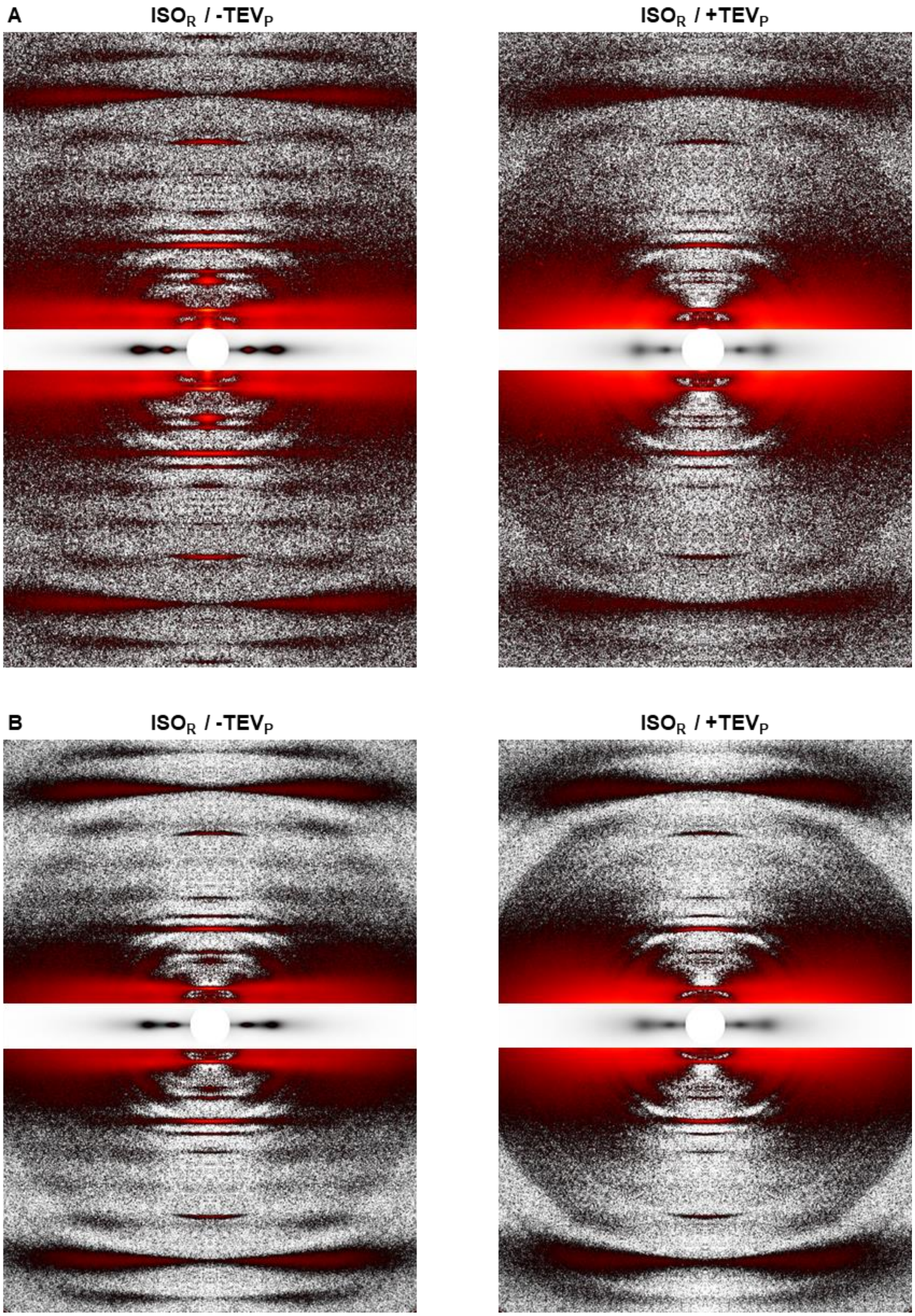

Fig. S5. Representative X-ray patterns during ISOR. (*A*) X-ray diffraction patterns from a single sample during IsoR both before (-TEVP) and after (+TEVP) TEV protease treatment. As previously indicated in passive muscle (22), 50% titin cleavage reduces image intensity but features of interest are still present. (*B*) A second representative example. For each sample, image intensities are shown as a heat map and are scaled across both images. For the equatorial intensities, the images were rescaled to allow for visualization of the equatorial reflections.

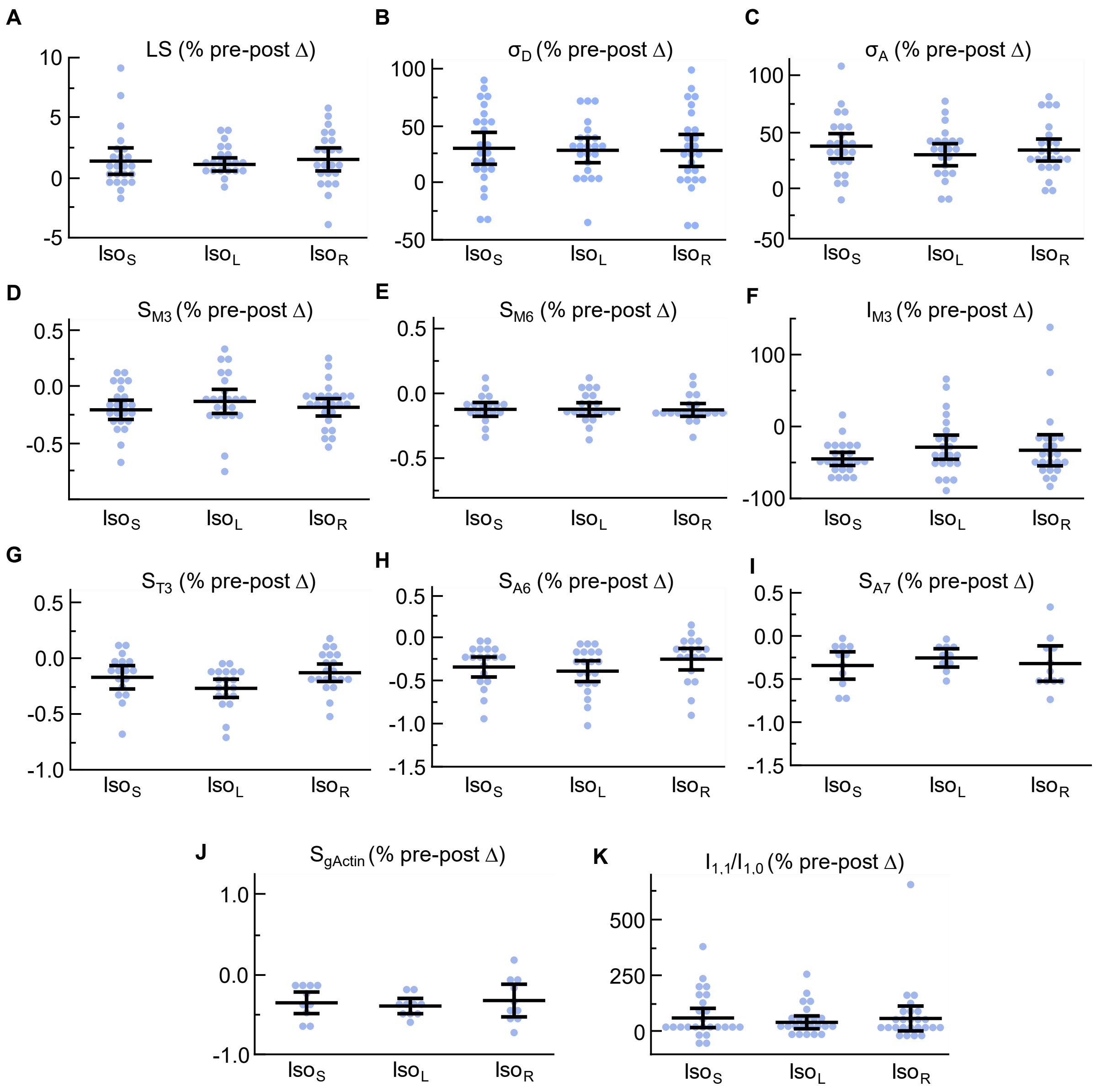

Fig. S6. Differences in parameters recorded by X-ray diffraction from mechanical experiments after 50% titin cleavage. (*A*-*K*) The post values for each X-ray diffraction parameter normalized to the paired pre-values. Data shown as mean ± 95% confidence interval of the mean.

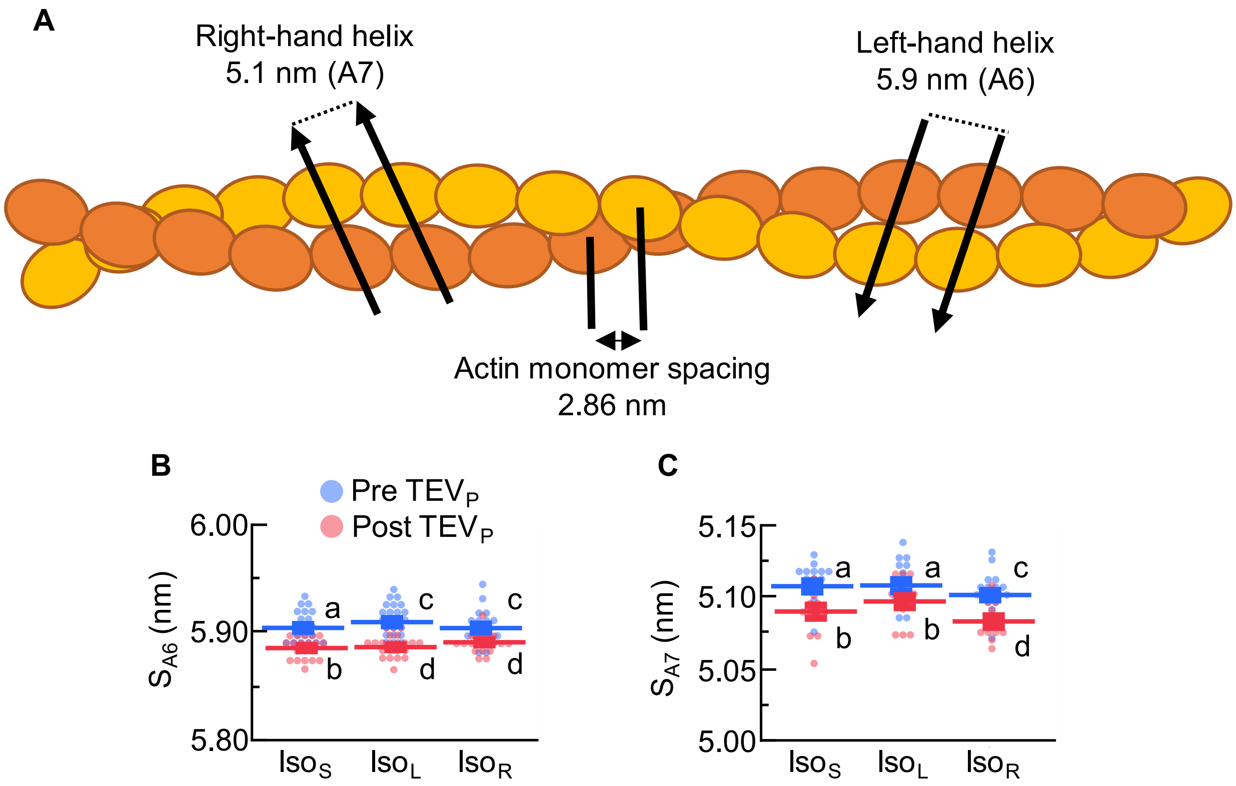

Fig. S7. Thin filament strain. (*A*) The actin double helix provides two repeating structures that are resolvable in our diffraction patterns, the right-handed helix (~5.1 nm repeat; S_{A7}), and the left-hand helix (~5.9 nm repeat; S_{A6}). The axial spacing of individual actin monomers (S_{gActin}, 2.86 nm) can be estimated with an equation (see methods). We provide spacing data for S_{A6} (n = 19-23) (*B*) and S_{A7} (n = 13-18) (*C*). The resulting actin monomer spacing, S_{gActin} is presented in Fig. 2I. Connecting letters: different letters are significantly different (Tukey HSD $P < 0.05$). Data shown as mean ± s.e.m. Statistical details in Table S3.

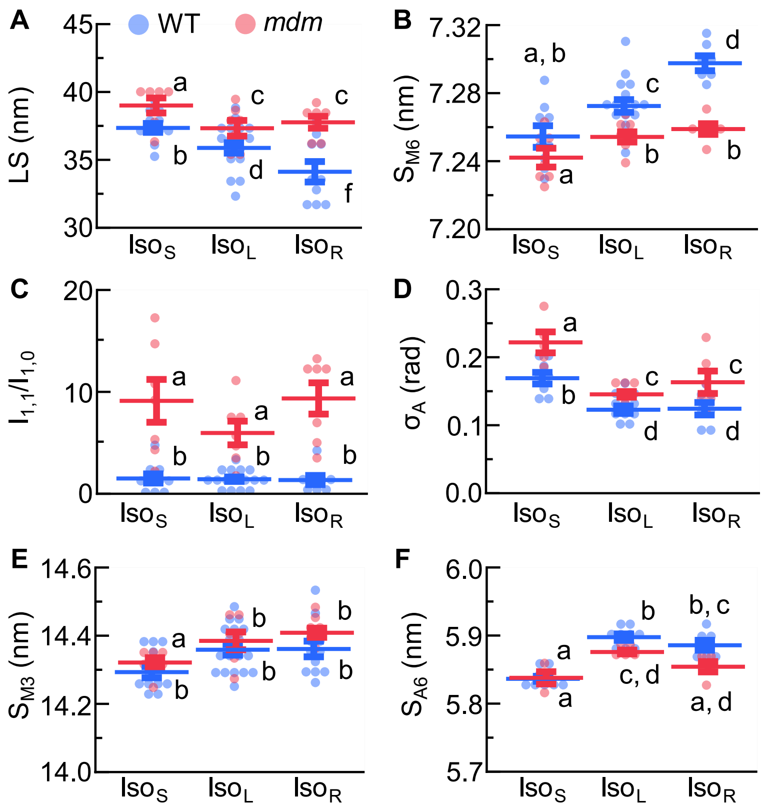

Fig. S8. Titinopathic *mdm* fibers produce no distinctive RFE condition. $D_{1,0}$ (*n* =7-17) (*A*), S_{M6} (*n* = 6-16) (*B*), $I_{1,1}/I_{1,0}$ (*n* = 7-16) (*C*), σ_A (*n* = 5-12) (*D*), S_{M3} (*n* = 7-18) (*E*), and S_{A6} (*n* = 4-9) (*F*) were recorded for WT (blue) and *mdm* (red) EDL fiber bundles at three conditions: Iso_S, Iso_L, and Iso_R. Statistics: ANOVA design with random effect of individual, followed by Tukey HSD multiple comparison procedure on significant main effects ($P < 0.05$). Data displayed as connecting letters: different letters are significantly different (Tukey HSD $P < 0.05$). Full statistical details in Table S4.

Table S1. Mechanics dataset, before and after TEV protease treatment (normalized to the active tension at Pre Iso_{Short}), from data in Figure 1. The ANOVA analysis F-stats and P-values are provided, as well as a connecting letter report from a Tukey HSD analysis. Data reported as mean ± s.e.m. *Significant (P < 0.05).

Parameter (n)	Condition	Mean ± s.e.m.	ANOVA Main Effects	F	P	Connecting Letters
**Passive Tension (28)**	Pre Iso_S	0.35 ± 0.03	Treatment	7.22	0.009*	a
**Passive Tension (24)**	Post Iso_S	0.23 ± 0.02	Condition	159.11	<0.0001*	b
**Passive Tension (28)**	Pre Iso_L	0.78 ± 0.08	Interaction	0.46	0.46	c
**Passive Tension (24)**	Post Iso_L	0.71 ± 0.08				d
**Active Tension (28)**	Pre Iso_S	1	Treatment	160.09	<0.0001*	a
**Active Tension (23)**	Post Iso_S	0.64 ± 0.03	Condition	54.72	<0.0001*	b
**Active Tension (27)**	Pre Iso_L	0.73 ± 0.03	Interaction	13.33	<0.0001*	c
**Active Tension (24)**	Post Iso_L	0.61 ± 0.04				b
**Active Tension (27)**	Pre Iso_R	1.04 ±0.04				a
**Active Tension (23)**	Post Iso_R	0.78 ± 0.04				c
**Total Tension (28)**	Pre Iso_S	1.35 ± 0.03	Treatment	145.70	<0.0001*	a,b
**Total Tension (23)**	Post Iso_S	0.87 ± 0.03	Condition	131.82	<0.0001*	c
**Total Tension (27)**	Pre Iso_L	1.49 ± 0.06	Interaction	10.90	<0.0001*	d
**Total Tension (24)**	Post Iso_L	1.31 ± 0.06				b
**Total Tension (27)**	Pre Iso_R	1.80 ± 0.08				e
**Total Tension (23)**	Post Iso_R	1.49 ± 0.08				a,c
**Passive Stiffness (31)**	Post Iso_S	0.72 ± 0.02	Condition	13.41	0.0007*	a
**Passive Stiffness (12)**	Post Iso_L	0.84 ± 0.02				b

Table S2. Changes in X-ray reflection data after TEV protease treatment, from data in Figure 2. The ANOVA analysis F-stats and P-values are provided, as well as a connecting letter report from a Tukey HSD analysis. Data reported as mean ± s.e.m. * P < 0.05.

Feature (n)	Treatment, Condition	Mean ± s.e.m.	Main Effects	F	P	Connecting Letters
LS (27)	Pre Iso_S	38.99 ± 0.26	Treatment	55.92	<0.0001*	a
LS (25)	Post Iso_S	40.02 ± 0.32	Condition	33.23	<0.0001*	b
LS (26)	Pre Iso_L	38.10 ± 0.22	Interaction	0.06	0.94	c
LS (24)	Post Iso_L	39.06 ± 0.20				d
LS (25)	Pre Iso_R	37.69 ± 0.21				e
LS (25)	Post Iso_R	38.57 ± 0.22				f
σ_A (26)	Pre Iso_S	0.13 ± 0.005	Treatment	183.13	<0.0001*	a
σ_A (24)	Post Iso_S	0.18 ± 0.01	Condition	2.49	0.09	b
σ_A (25)	Pre Iso_L	0.14 ± 0.01	Interaction	1.00	0.37	a
σ_A (23)	Post Iso_L	0.17 ± 0.01				b
σ_A (24)	Pre Iso_R	0.13 ± 0.01				a
σ_A (24)	Post Iso_R	0.17 ± 0.01				b
σ_D (25)	Pre Iso_S	9.69 ± 0.37	Treatment	30.39	<0.0001*	a
σ_D (23)	Post Iso_S	10.85 ± 0.41	Condition	1.20	0.30	b
σ_D (24)	Pre Iso_L	10.07 ± 0.30	Interaction	0.13	0.88	a
σ_D (22)	Post Iso_L	11.18 ± 0.26				b
σ_D (23)	Pre Iso_R	9.87 ± 0.34				a
σ_D (23)	Post Iso_R	11.19 ± 0.41				b
$I_{1,1}/I_{1,0}$ (27)	Pre Iso_S	1.37 ± 0.07	Treatment	24.7364	<0.0001*	a
$I_{1,1}/I_{1,0}$ (25)	Post Iso_S	2.25 ± 0.33	Condition	1.7432	0.18	b
$I_{1,1}/I_{1,0}$ (26)	Pre Iso_L	1.47 ± 0.06	Interaction	0.0773	0.93	a
$I_{1,1}/I_{1,0}$ (24)	Post Iso_L	2.12 ± 0.24				b
$I_{1,1}/I_{1,0}$ (25)	Pre Iso_R	1.47 ± 0.07				a
$I_{1,1}/I_{1,0}$ (25)	Post Iso_R	2.32 ± 0.31				b
S_{M3} (26)	Pre Iso_S	14.43 ± 0.01	Treatment	60.671	<0.0001*	a
S_{M3} (24)	Post Iso_S	14.40 ± 0.01	Condition	0.788	0.457	b
S_{M3} (26)	Pre Iso_L	14.43 ± 0.01	Interaction	0.746	0.477	c
S_{M3} (24)	Post Iso_L	14.41 ± 0.01				d
S_{M3} (26)	Pre Iso_R	14.44 ± 0.01				c
S_{M3} (25)	Post Iso_R	14.41 ± 0.01				d
S_{M6} (22)	Pre Iso_S	7.244 ± 0.001	Treatment	59.15	<0.0001*	a
S_{M6} (20)	Post Iso_S	7.233 ± 0.003	Condition	12.02	<0.0001*	b
S_{M6} (25)	Pre Iso_L	7.247 ± 0.002	Interaction	0.13	0.88	c
S_{M6} (21)	Post Iso_L	7.239 ± 0.002				d
S_{M6} (24)	Pre Iso_R	7.252 ± 0.002				e
S_{M6} (20)	Post Iso_R	7.242 ± 0.002				f

Table S3. Changes in X-ray reflection data after TEV protease treatment, from data in Figure 2. The ANOVA analysis F-stats and P-values are provided, as well as a connecting letter report from a Tukey HSD analysis. Data reported as mean ± s.e.m. * P < 0.05.

Feature (n)	Treatment, Condition	Mean ± s.e.m.	Main Effects	F	P	Connecting Letters
S_{T3} (24)	Pre Iso_S	12.74 ± 0.005	Treatment	52.55	<0.0001*	a
S_{T3} (19)	Post Iso_S	12.73 ± 0.01	Condition	7.69	0.001*	b
S_{T3} (23)	Pre Iso_L	12.77 ± 0.004	Interaction	3.15	0.047*	c
S_{T3} (21)	Post Iso_L	12.73 ± 0.01				a, b
S_{T3} (22)	Pre Iso_R	12.75 ± 0.01				a
S_{T3} (20)	Post Iso_R	12.73 ± 0.01				a, b
S_{A6} (19)	Pre Iso_S	5.918 ± 0.002	Treatment	92.21	<.0001	a
S_{A6} (21)	Post Iso_S	5.900 ± 0.003	Condition	1.98	0.14	b
S_{A6} (23)	Pre Iso_L	5.923 ± 0.002	Interaction	1.17	0.22	c
S_{A6} (20)	Post Iso_L	5.902 ± 0.003				d
S_{A6} (19)	Pre Iso_R	5.920 ± 0.003				c
S_{A6} (20)	Post Iso_R	5.906 ± 0.004				d
S_{A7} (15)	Pre Iso_S	5.107 ± 0.004	Treatment	49.89	<0.0001*	a
S_{A7} (13)	Post Iso_S	5.089 ± 0.004	Condition	9.79	0.0002*	b
S_{A7} (15)	Pre Iso_L	5.108 ± 0.004	Interaction	0.88	0.42	a
S_{A7} (14)	Post Iso_L	5.097 ± 0.004				b
S_{A7} (18)	Pre Iso_R	5.101 ± 0.003				c
S_{A7} (13)	Post Iso_R	5.083 ± 0.004				d
S_{gActin} (14)	Pre Iso_S	2.738 ± 0.001	Treatment	99.92	<0.0001*	a
S_{gActin} (12)	Post Iso_S	2.729 ± 0.001	Condition	5.75	0.005*	b
S_{gActin} (15)	Pre Iso_L	2.741 ± 0.001	Interaction	0.06	0.94	c
S_{gActin} (14)	Post Iso_L	2.732 ± 0.001				d
S_{gActin} (16)	Pre Iso_R	2.737 ± 0.001				a
S_{gActin} (12)	Post Iso_R	2.728 ± 0.001				e
I_{M3} (26)	Pre Iso_S	1	Treatment	98.16	<0.0001*	a
I_{M3} (24)	Post Iso_S	0.54 ± 0.04	Condition	2.43	0.093	c
I_{M3}(26)	Pre Iso_L	0.79 ± 0.05	Interaction	3.53	0.032*	b
I_{M3} (24)	Post Iso_L	0.56 ± 0.06				c
I_{M3} (26)	Pre Iso_R	0.99 ± 0.09				a, b
I_{M3} (24)	Post Iso_R	0.54 ± 0.04				c

Table S4. X-ray reflection data between WT and *mdm* muscle, from data in Figure 3. The ANOVA analysis F-stats and P-values are provided, as well as a connecting letter report from a Tukey HSD analysis. Data reported as mean ± s.e.m. * P < 0.05.

Parameter (n)	Genotype, Condition	Mean ± s.e.m.	ANOVA Main Effects	F	P	Connecting Letters
LS (11)	WT Iso_S	37.35 ± 0.33	Genotype	16.15	0.0006*	a
LS (7)	*mdm* Iso_S	39.01 ± 0.55	Condition	14.92	<0.0001*	b
LS (17)	WT Iso_L	35.88 ± 0.39	Interaction	3.80	0.032*	c
LS (7)	*mdm* Iso_L	37.33 ± 0.58				a
LS (10)	WT Iso_R	34.12 ± 0.75				d
LS (7)	*mdm* Iso_R	37.77 ± 0.44				a, b
σ_A (8)	WT Iso_S	0.17 ± 0.01	Genotype	9.57	0.007*	a
σ_A (5)	*mdm* Iso_S	0.22 ± 0.02	Condition	37.79	<0.0001*	b
σ_A (12)	WT Iso_L	0.12 ± 0.01	Interaction	0.77	0.47	b
σ_A (5)	*mdm* Iso_L	0.15 ± 0.004				
σ_A (7)	WT Iso_R	0.12 ± 0.01				
σ_A (6)	*mdm* Iso_R	0.16 ± 0.02				
$I_{1,1}/I_{1,0}$ (10)	WT Iso_S	1.50 ± 0.45	Genotype	39.80	<0.0001*	a
$I_{1,1}/I_{1,0}$ (7)	*mdm* Iso_S	9.12 ± 2.10	Condition	1.99	0.1535	b
$I_{1,1}/I_{1,0}$ (16)	WT Iso_L	1.43 ± 0.22	Interaction	2.27	0.1203	a
$I_{1,1}/I_{1,0}$ (7)	*mdm* Iso_L	5.96 ± 1.16				b
$I_{1,1}/I_{1,0}$ (8)	WT Iso_R	1.35 ± 0.45				a
$I_{1,1}/I_{1,0}$ (7)	*mdm* Iso_R	9.35 ± 1.53				b
S_{M3} (12)	WT Iso_S	14.29 ± 0.02	Genotype	2.35	0.1413	a
S_{M3} (7)	*mdm* Iso_S	14.32 ± 0.01	Condition	12.45	<.0001	b
S_{M3} (18)	WT Iso_L	14.36 ± 0.02	Interaction	0.32	0.7297	b
S_{M3} (7)	*mdm* Iso_L	14.38 ± 0.03				
S_{M3} (12)	WT Iso_R	14.36 ± 0.02				
S_{M3} (7)	*mdm* Iso_R	14.40 ± 0.01				
S_{M6} (9)	WT Iso_S	7.25 ± 0.006	Genotype	16.17	0.0007*	a, b
S_{M6} (7)	*mdm* Iso_S	7.24 ± 0.006	Condition	34.92	<0.0001*	a
S_{M6} (16)	WT Iso_{Lo}	7.27 ± 0.004	Interaction	8.29	0.0015*	b
S_{M6} (7)	*mdm* Iso_L	7.25 ± 0.003				c
S_{M6} (7)	WT Iso_R	7.30 ± 0.004				d
S_{M6} (6)	*mdm* Iso_R	7.26 ± 0.003				b
S_{A6} (9)	WT Iso_S	5.84 ± 0.003	Genotype	10.27	0.007*	a
S_{A6} (4)	*mdm* Iso_S	5.84 ± 0.009	Condition	36.09	<0.0001*	a
S_{A6} (9)	WT Iso_L	5.90 ± 0.005	Interaction	3.97	0.03*	b
S_{A6} (5)	*mdm* Iso_L	5.88 ± 0.002				c, d
S_{A6} (7)	WT Iso_R	5.89 ± 0.007				b, c
S_{A6} (5)	*mdm* Iso_R	5.85 ± 0.002				a, d

Dataset S1. Source_Data (Separate Excel file)

All data used to generate figures and conduct statistical analysis are provided.
